# Supplementary material for: Leveraging vibration of effects analysis for robust discovery in observational biomedical data science
Source: PLoS Biol. 2021 Sep 23;19(9):e3001398. doi: 10.1371/journal.pbio.3001398 (PMC8510627; doi:10.1371/journal.pbio.3001398)
Supplement: Attachment — Submitted filename: quantvoe_response_to_reviewers_second_round.pdf [file pbio.3001398.s010.pdf]

To the Editors and Reviewers,

We once again thank you for your consideration of and comments on our manuscript. We were thrilled to find that our changes were to your liking, and we agree that they substantially improved the manuscript.

We have now updated the text according to the most recent comments, which, as before, we reproduce below in a point-by-point response in blue. Also as before, we have provided a tracked-changes manuscript.

We once again thank you for your time and look forward to your response.

Sincerely, and on behalf of the authors,

Chirag J Patel

Editor Comments:

You may be aware of the PLOS Data Policy, which requires that all data be made available without restriction: http://journals.plos.org/plosbiology/s/data-availability. For more information, please also see this editorial: http://dx.doi.org/10.1371/journal.pbio.1001797

Note that we do not require all raw data. Rather, we ask that all individual quantitative observations that underlie the data summarized in the figures and results of your paper be made available in one of the following forms:

1) Supplementary files (e.g., excel). Please ensure that all data files are uploaded as 'Supporting Information' and are invariably referred to (in the manuscript, figure legends, and the Description field when uploading your files) using the following format verbatim: S1 Data, S2 Data, etc. Multiple panels of a single or even several figures can be included as multiple sheets in one excel file that is saved using exactly the following convention: S1_Data.xlsx (using an underscore).

We have renamed the files according to this structure, additionally changing how we reference them in the text.

2) Deposition in a publicly available repository. Please also provide the accession code or a reviewer link so that we may view your data before publication.

Regardless of the method selected, please ensure that you provide the individual numerical values that underlie the summary data displayed in the following figure panels as they are essential for readers to assess your analysis and to reproduce it: Figure 4ACEG.

We have deposited the raw modeling output on Figshare and made it publicly available. The analysis carried out in the manuscript for the figures is captured in the two items contained within this directory. We state in the project repository that the data in the Figshare folder can be used, in conjunction with linked code in our GitHub repository, to recreate the analysis and figures from the manuscript.

https://figshare.com/projects/quantvoe_manuscript_analysis_files/120969

3) Please also ensure that figure legends in your manuscript include information on where the underlying data can be found, and ensure your supplemental data file/s has a legend.

We have confirmed that all 4 supplementary data files have a legend, and we now reference where the raw data can be found for the data contained in the other figures.

4) Please ensure that your Data Statement in the submission system accurately describes where your data can be found.

We have updated the Data Statement accordingly, specifically adding in a reference to our GitHub repository. We write:

"The software developed for this project, including scripts used to build the NHANES datasets, is available at https://github.com/chiragjp/quantvoe. The output of the quantvoe analysis, which can be used in conjunction with https://github.com/chiragjp/quantvoe/blob/main/manuscript/voe_pipeline_figs.Rmd to generate the figures in this manuscript, has been deposited at https://figshare.com/projects/quantvoe_manuscript_analysis_files/120969. The UK Biobank data is available upon request from https://www.ukbiobank.ac.uk/. The UK Biobank project number for this project was 22881."

5) As you address these items, please take this last chance to review your reference list to ensure that it is complete and correct. If you have cited papers that have been retracted, please include the rationale for doing so in the manuscript text, or remove these references and replace them with relevant current references. Any changes to the reference list should be mentioned in the cover letter that accompanies your revised manuscript.

We reviewed the reference list and were unable to find any retracted manuscripts. The only changes we made were the addition of the reference mentioned by Reviewer 1 in point 6, and the removal of a reference to the correlation between chocolate consumption and the Nobel Prize that, while it was no longer referenced in the text per a previous reviewer comment, slipped through into the latest reference list version.

6) Reviewer #1: This manuscript is much improved as is the Github repository. There are still some issues with the software but as these might be system dependent I will move these comments to issues on Github. The README is clear and comprehensive, indeed, one of the best I have seen. I was able to run the 'beginner' example with no problems. There are still grammatical type setting issues which I have sent separately in a marked up word document to the editor.

I recommend this for publication with the following minor revision: re: Janus effect - some reference to the literature on sign changes in regression modelling would be beneficial, e.g., doi: 10.1186/1742-7622-5-5

We are thrilled that the Reviewer found our manuscript improved, and we are very grateful for their additional comments. Their comments on the README are particularly flattering -- thank you. We look forward to making further improvements to the software in GitHub as needed.

Regarding the Janus Effect -- we have integrated that reference into the text and mentioned in the discussion this point regarding the potential benefit of sign switching. We write:

"It is additionally worth noting that when signs change sign, or the Janus Effect, across model specifications is neither entirely unexpected (due to the underlying correlation structure of the

data) nor necessarily a negative(Arah 2008) with respect to overall findings. In the examples we describe, the Janus Effect could help identify potential confounding variables."

7) Reviewer #2: I would like to thank the authors for revising their paper. I think that the paper is in a better shape now and I recommend it for publication.

We thank the Reviewer for this comment and are pleased they found it suitable for publication.

8) Reviewer #3: The authors have well addressed the points raised in the previous review and the relation of the proposed technique with others is now well described.

One remaining point:

I am a little confused by the use of the terms "highly discordant" in the abstract :
77.3% [associations] were highly discordant, with greater than 5%"5% of all models generating conflicting (i.e. positive vs. negative) results"
Given the possible correlations between model variables, one would actually expect some negative associations.

I would therefore specify that this is not an unexpected result and only indicates the importance of the model selection, and reinforce the importance of the use of classical model selection techniques in the discussion.

We thank the Reviewer for their comment and have sought to address it in the text. Specifically, we reworded the abstract slightly to emphasize that we were exploring why the sign was switching, reducing the hyperbole resulting from the use of the phrase "highly discordant" and clarifying that one purpose of our analysis was to explore the underlying source of conflicting model associations. We write:

"We execute a VoE analysis on a series of widely reported associations (e.g. carrot intake and eyesight) with an extended additional focus on lifestyle exposures (e.g. physical activity) and components of the Framingham Risk Score for cardiovascular health (e.g. blood pressure). We leveraged our tool for potential confounder identification, investigating what adjusting variables are responsible for conflicting models."

This comment is specifically similar to Reviewer 1's remaining comment above. We hope that this reference (recommended by Reviewer 1) that we added to the text, as well as a brief description of the possible benefit/expected nature of sign switching in the discussion, addresses this final concern.

To reiterate, we now write in the Discussion:

"It is additionally worth noting that when signs change sign, or the Janus Effect, across model specifications is neither entirely unexpected (due to the underlying correlation structure of the

data) nor necessarily a negative(Arah 2008) with respect to overall findings. In the examples we describe, the Janus Effect could help identify potential confounding variables.”
