# Supplementary material for: Leveraging vibration of effects analysis for robust discovery in observational biomedical data science
Source: PLoS Biol. 2021 Sep 23;19(9):e3001398. doi: 10.1371/journal.pbio.3001398 (PMC8510627; doi:10.1371/journal.pbio.3001398)
Supplement: Attachment — Submitted filename: response_to_editors.docx [file pbio.3001398.s007.docx]

4/5/2021

To Dr. Roland Roberts and the PLOS Biology Editorial team,

We thank you for your consideration of a previous version of our manuscript (reference number PBIOLOGY-D-21-00794, originally submitted on 3/22/2021) and for you and the associate editors’ constructive feedback. We agree with the comments that our work required further comparison to the literature (specifically multiverse analysis) and was too dense. We have aimed to remedy both of these problems in our revision, and we believe the result has been a much improved and streamlined manuscript. We respond to each of the Academic Editor’s points specifically below in blue, and we have provided a tracked changes version of the manuscript in our new submission.

In brief, we:

1) Removed extraneous jargon from the entire manuscript and made the introduction more relevant to a broad audience,
2) Relegated the dense first section of our manuscript regarding benchmarking our algorithm to supplemental text,
3) Placed our tool in the broader literature of multiverse and sensitivity analysis (including those recommended by the editor)

Overall, we believe that incorporating the editor’s recommendations has made clear both the importance of our work to the scholarly community as well as the broader public. Once again, thank you for your consideration and we look forward to your response.

Sincerely, and on behalf of the authors,

Chirag J Patel, PhD

Associate Professor of Biomedical Informatics

Harvard Medical School

Academic Editor's concerns:

a) The Academic Editor sees your approach as effectively a multiverse analysis. Although your computational tool is potentially powerful, the AE feels that the basic idea is not itself novel: https://journals.sagepub.com/doi/10.1177/1745691616658637 and another similar approach: https://papers.ssrn.com/sol3/papers.cfm?abstract_id=2694998 and an application here: https://www.nature.com/articles/s41562-018-0506-1 Before review, you would therefore need to clearly articulate what is novel about your approach relative to, for example, multiverse analysis (and cite these other related examples in the Introduction).

We agree with the editor that our work is an automated implementation of one type of multiverse analysis. We now extensively address how our manuscript fits into this broader framework, writing in the introduction:

“Modeling VoE can be thought of as a specific type of “multiverse analysis” designed a “discovery” scenario in biomedical sciences, where there are many possible X variables to be correlated with a Y variable, such as metagenomic-phenotype association study or exposome-wide association study.[(Forslund et al. 2015; Patel et al. 2010)](https://paperpile.com/c/wNIKR0/ZvcZ+NqE7) Multiverse analyses, very broadly speaking, describe how any analytic choice (e.g. data preparation, model assumptions, model type) can change findings[(Steegen et al. 2016; Orben and Przybylski 2019)](https://paperpile.com/c/wNIKR0/mrgf+irJz). But few of the many possible types of multiverse analysis have been fully automated and many require manual configuration. Additionally, some of these tools, such as SpecificationCurve Analysis[(Simonsohn et al. 2019; Simonsohn et al. 2020)](https://paperpile.com/c/wNIKR0/nqio+OreQ), rely on users “enumerating all data analytic decisions necessary to map a hypothesis onto a set of possible associations”, a step often not feasible for complex, discovery-based questions and analyses, such as genomics. VoE expands on multiverse analysis, fully automating the exploration of model choice. For example, we have recently used VoE modeling to prioritize >1 million correlations in metagenomic-phenotype associations (Tierney et al, Nature Comm in press).”

“Other work and software packages for modeling sensitivity under the umbrella of multiverse analysis is additionally focused less on modeling strategy and more on alternative aspects of data analysis, such as data collection.(Harder 2020) Finally, there is a need also missing from these other approaches to not just demonstrate how results change as a function of approach, but specifically why they do: what adjusters confound associations the most and therefore should always be considered in future studies.”

And in the discussion, we specifically remind readers that our work falls in the framework of multiverse analysis, writing:

“Indeed, many “popular” associations -- like the association between egg consumption and mortality,[[34–36]](https://paperpile.com/c/wNIKR0/0YSGW+7pNoI+41u7l) the association between wine consumption and cardiovascular health,[[37,38]](https://paperpile.com/c/wNIKR0/4KAzj+AlW3N) and even the results of famous “marshmallow experiment” as an indicator of a child’s self-control and success later in life[[39–42]](https://paperpile.com/c/wNIKR0/fI60O+1cTYs+GxfkF+Tuyv8) have conflicting reports of directionality that, we hypothesize, could potentially be attenuated by multiverse analyses such as probing the space of VoE possible for those variables.”

b) The Academic Editor also asks you to make your manuscript less dense and more accessible. PLOS Biology is a general biology journal with a broad readership, and you should aim to maximise the appeal of your paper not only to its direct target market, but also those in related fields who might find its conclusions and methods useful.

We thank the Editor for this comment and agree that our initial manuscript was technical to the point of being dense. We have made a number of changes to the text, including reordering and rewriting entire sections to effectively communicate our work’s relevance to the broad readership of PLOS Biology.

Specifically:

1) We adjusted the beginning of the introduction so that we open with clear examples of popularized associations computed across a number of biological fields (e.g. chocolate consumption and nobel prizes, the human microbiome and disease) to demonstrate how truly all-encompassing observational data science and the problems facing it really are.
2) Added extensive text modifications to describe how the novelty of our approach lies in automating a previously underexplored and non-programmatic aspect of multiverse analysis (modeling Vibration of Effects and exploring the root cause of Janus Effects). We additionally clarify that our work is particularly relevant to “discovery” or hypothesis generation in a “big data” setting.
3) We additionally cleaned up the text throughout the manuscript, removing convoluted phrasing and complicated jargon (e.g. excessive references to sensitivity analysis or janus effects when other language would work just as well).
4) We took what was the first section of the results (describing the optimal number of vibrations needed to fully explore a dataset) and moved it to the end, as we felt it better to ground readers by immediately launching into our “example” associations, instead of starting with a highly technical dive into algorithmic performance and best practices.
