# Supplementary material for: Leveraging vibration of effects analysis for robust discovery in observational biomedical data science
Source: PLoS Biol. 2021 Sep 23;19(9):e3001398. doi: 10.1371/journal.pbio.3001398 (PMC8510627; doi:10.1371/journal.pbio.3001398)
Supplement: Attachment — Submitted filename: quantvoe_response_to_reviews.docx [file pbio.3001398.s009.docx]

To the Editors and Reviewers,

We thank you for the comments on our manuscript and for taking the time to make them -- we have revised our work to integrate them and find the new product to be much improved.

Very broadly speaking, we:

1. Emphasize the novelty of our manuscript lying in the development of a software tool to operationalize modeling Vibration of Effects.
2. Extended our unit-testing and software examples in our code while also drastically expanding the README to clarify the details of using and testing quantvoe.
3. We systematically reworked the Discussion and Introduction to address Reviewer comments regarding rigorously defining terms and stating potential drawbacks and extensions of our approach.

We provide point by point response to reviewer comments below in blue, citing passages from the text where relevant. Where we wish to point out specific changes to the text that are intermingled with unchanged text, we highlight these key phrases in yellow. As requested, we provide a tracked-changes manuscript and also reproduce the entirety of Reviewer comments (while adding in a numbering scheme to make them easily referenceable).

We once again thank you for your time and look forward to your response.

Sincerely and on behalf of the authors,

Chirag J Patel

**Reviewer #1:**

**[identifies himself as Robert E. Arbon]**

This paper presents a tool for performing a type of multiverse analysis known as a vibration of effects (VoE). This tool is applicable in the situation where one is interested in modelling the effect of X on Y but where there are a large number of potential confounders and no underlying theory to inform the modelling strategy. The tool, quantvoe, aims at "automatically identifying what measured adjusting variables drive inconsistency in associations between X and Y". It does this by modelling a wide range of model specifications and then modelling the impact of adjustments on the size of a measured association using a linear (potentially multilevel) model.

As already noted in the editor's comments, the approach of VoE and multiverse analysis is not novel but developing a robust tool to perform this analysis is worthy contribution the field. The approach to identifying variables which affect associations is an interesting and novel one.

The significance of the work is hard to judge (see revisions below) and the authors need to make it very clear how this tool is to be used in a practical research sense.

I recommend this paper for publication, once a number of major revisions have been made.

We thank the Reviewer for their comments and are thrilled that they are enthusiastic enough about our work to eventually recommend its publication and that it is a worthy contribution to the field.

Major revisions:

1.1.	The title of the paper does not match the content and one of them should change (preferably the content). It is not clear how to use the output of the quantvoe to inform further research. You mention in passing (71 - 73) using VoE to prioritise correlations, please expand on this, or develop the examples given in the same manner, even if they are hypothetical situations. A figure summarising quantvoe's place in the broader research workflow would be an excellent addition but not necessary.

We agree with the Reviewer that the content of our paper should be more clearly related to our title. We have now updated the Introduction and Discussion to achieve this end. We reproduce these changes below:

In the Introduction:

“Charting a course through the process of analyzing observational data (Fig 1) is not always straightforward, however. Any decision along the analytic pipeline -- from data collection to statistical analysis -- can introduce uncertainty (e.g. false positives), obscuring underlying biology.[5–7] As a result, there is a large need for methods to aid in navigating the maelstrom of the analytic process. Here, we present a tool, “quantvoe,” for mitigating one of many potential biases: confounding via model specification. Broadly speaking, quantvoe operationalizes the identification of robust (i.e. high-confidence) vs. non-robust (i.e. low-confidence) associations, enabling greater long-term model reproducibility and interpretability. For example, if our tool had existed in the past year, we could have understood one aspect of the confusing and contradictory claims regarding COVID-19 positivity and Vitamin D levels.(Rubin 2021)”

“...Other work and software packages for modeling sensitivity under the umbrella of multiverse analysis is additionally focused less on modeling strategy and more on alternative aspects of data analysis, such as data collection.[[20]](https://paperpile.com/c/FNAJOA/uhOG4) We propose this gap is limiting the ability of researchers to navigate observational data, identifying robust associations and potential confounders. Indeed, in other disciplines (e.g. genetics) the development of analogous software (e.g. PLINK (Purcell et al. 2007)) democratized genetic epidemiology and meta-analysis (e.g. via Genome Wide Association Studies [GWASs]). Finally, there is also a need also missing from these other approaches to not just quantify VoE demonstrate how results change as a function of approach, but specifically show why model output may vary why they do: what adjusters confound associations the most and therefore should be considered in future studies.”

In the Discussion:

“While VoE has been demonstrated before, it has remained hitherto inaccessible to analysts due to the complexity of its requisite analyses. The novelty of our tool -- quantvoe -- lies in its ability to provide software to efficiently automate the bridging the gap between data and estimation of associations in observational studies. By using quantvoe, researchers can identify which associations are robust (or not) and why this is the case prior to publishing them or pursuing them in future experiments.”

“Overall, we have developed software for automatically assessing vibration of effects to identify adjusting variables that may foul up associations, leading investigators and the public astray. This software allows researchers to automate sensitivity analyses, or Vibration of Effects, to navigate through large-scale association studies, fulfilling a tenet of biomedical data science: computing robust associations. We used...”

We additionally have, per the Reviewer’s request, created a figure demonstrating quantvoe’s in an analytic workflow. We reproduce this Figure (part of Figure 1) and its legend below.

Figure 1: **Overview of the algorithmic approach and its place in the hypothesis generation toolkit.** A) The process/paradigm of “data-driven” discovery. First, high-dimensional data is collected on a set of individuals with a given phenotype (i.e. a disease) as well as controls (individuals without the phenotype). The researcher selects a modeling strategy, most often just one, and computes associations between the phenotype of interest (a “Y”) and a particular feature (an “X,” like coffee in the example). The resultant associations yield associational “hypotheses” regarding the relationship between the X and the Y, but only fitting one model specification can yield non-robust results. Quantvoe replaces this middle step of choosing one model, instead attempting to fit up to every possible model specification, thereby charting a course to robust hypothesis generation. B) Quantvoe takes three types of input data, all in the form of pairs of data frames (tables), either at the command line or in an interactive R session: 1) a single dependent variable and multiple independent variables, 2) multiple dependent variables, or 3) multiple datasets. C) There are 4 main steps -- checking the input data, computing initial univariate associations, computing vibrations across possible adjusters, and quantifying how adjuster presence/absence correlates to changes in the primary association of interest. D) Following computing VoE, we evaluate the results by measuring Janus Effect (the fraction of associations greater than 0) and estimating the impact of different adjusters on the change in correlation size.

1.2.	In the same vein as point 1, please provide some indication as to what the Janus effect actually means in practice.

We now clarify what a Janus effect practically means in the following updated passage in the Introduction:

“It is capable of identifying highly non-robust associations with “Janus Effects” (JE), where model specification yields contradicting results, reporting positive and negative output. In practice, JEs can be used to understand if an association between two variables is consistent regardless of model specification.”

1.3.	I was not able to follow the instructions for the tests of quantvoe (on the Github repo) as the necessary datasets were not provided. Please provide this so this software can be tested. Alternatively, please provide detailed instructions/commands to run with the NHANES dataset provided.

We agree that our initial testing suite was unclear and insufficient, as both Reviewers 1 and 3 pointed out. As a result, we have expanded and clarified our package testing approach.

In total, our pipeline can be checked and validated using 3 different strategies which employ different metrics: 1) a package vignette, 2) a unit testing suite, and 3) example command line deployments. We now indicate in the manuscript Methods the existence of tests and how they can be run. We write:

###### “Package installation and testing

The package can be downloaded from GitHub. Detailed installation instructions and other package details are located in the GitHub README. Quantvoe is replete with an example vignette to demonstrate use in the R terminal, unit testing suite, and example (bash) command line deployments.”

We will describe here the specific updates to the README and package that have made these easier to run for users and Reviewers.

Package vignette

Prior to describing the unit testing suite, the README now focuses on the R package vignette that was included with the initial package but not made clear in the documentation (it now has its own section within the Testing section of the README). We additionally did not make clear initially in the package installation instructions that, to access this vignette at the R terminal, the build_vignettes flag needed to be set to TRUE. We have remedied this error, and the README is updated accordingly.

This vignette demonstrates an example of how the pipeline would be run from the R terminal (as opposed to the command line implementation included in the GitHub repository). It describes the example datasets included in the package (as environmental variables upon loading library(quantvoe)), runs the full pipeline, and then provides examples as to how to interpret the output, including detailed plotting instructions.

If installed when the package is built with the build_vignettes=TRUE flag, the vignette can be accessed using:

browseVignettes(‘quantvoe’).

Alternatively, the .Rmd file that contains it can be accessed in the vignettes/ directory within the quantvoe package.

We have added a section to the README that describes how to run the vignette:

Unit testing suite

In the GitHub repository README, we clarified exactly how to run the tests. Previously our instructions were vague, at best. We now specify where datasets of interest are as well as what the actual tests run by the pipeline actually do. **At the bottom of this document, we include screenshots as to what successful a Unit testing run actually looks like.**

These updates can be seen here:

Command line deployment

First, we additionally realized, as the Reviewer pointed out, that there was an error in our documentation: the datasets used in the command line deployment were present NOT in the folder initially stated on the README. They were said to be in the data/ directory, in fact they were in the inst/extdata/ directory. We apologize for this oversight and have addressed the error.

Otherwise, other than a few minor changes to the text of the relevant section in the README and fixing the data location error, these tests are primarily unchanged. However, at the bottom of this document we provide examples of what the terminal output for each of the three examples should be.

1.4.	Please discuss alternative approaches to biomedical research in the same context i.e., when there is little or no theory and lots of measured variables. There are other types of sensitivity analysis e.g., VanderWeele's E values. Please compare these approaches to quantvoe using the examples given.

We have now explicitly discussed a variety of alternative sensitivity analyses in the Discussion. We write:

“There are of course additional many other methods that could be used for sensitivity analysis. A non-exhaustive list includes VanderWeele's E-values (which measures how strong the association between potential confounding variables, the outcome, and the exposure would have to be to influence an association of interest), Monte Carlo sensitivity analysis (which attempts to correct for bias based on model parameters), or the Confidence Profile Method (which is a Bayesian approach to measure uncertainty).[(VanderWeele and Ding 2017; Greenland 2005)](https://paperpile.com/c/GreDYQ/RbBt+zvWv). All of these methods have their strengths and limitations[(Ioannidis et al. 2019)](https://paperpile.com/c/R8uuSU/Vnaw). The key difference between these tools and VoE is that VoE explicitly models the impact of specific and measured adjusting variables or model specifications on an association size instead of using outside parameters and assumptions to do so. That said, these methods could be used to augment quantvoe, or potentially they could be deployed in its stead in situations where the potential drawbacks of the brute force modeling approach outweigh the benefits.”

Further, as the Reviewers point out, the novelty of this paper lies in operationalizing an existing framework -- modeling VoE. Therefore, while certainly a goal, we believe it is outside the scope of this manuscript to explicitly compare VoE to other forms of sensitivity analysis by running these different methods on our datasets.

1.5.	Please provide a justification for your claim:

…our package circumvents the need for a priori selection of a small set of candidate variables…

We have removed this claim and instead clarified how our package can be used to discover candidate adjusting variables, writing:

“Simply put, for a given dataset, our package fits up to every possible model using every covariate present and analyzing variation in the output (Fig 1). This analysis yields lists of candidate variables to potentially be considered in later analyses.”

1.6.	Please provide validation of the modelling approach for identifying important adjusting variables, i.e., equations 10 and 11 for some of the examples used. E.g., residual plots etc.

We agree that validation of the confounder analysis was missing from our initial manuscript and package.

First, for the analysis in Figure 4, we have now added this validation in the form of a supplementary figure describing regression summary statistics and plots. We reproduce the Figure, with it’s legend, below:

Supplementary Figure 1: Regression validation plots for example confounder analyses. Quadrant titles (e.g. Y ~ X) match the names in Figure 4.

We additionally write in the results:

“We additionally report model validation plots for this analysis (e.g. residuals) in Supplementary Figure 1.”

We additionally discuss these plots in the Discussion, encouraging users to view the confounder analysis as a “first pass” that should be explored with deeper rigor to confirm the models are satisfactory given the input variables. We additionally propose future changes to the software that could allow for other forms of confounder analysis.

We write:

“Additionally, our analysis to identify potential confounders (Fig 4) may have some drawbacks if the models violate some of the assumptions of regression. While, on average, the examples in this manuscript did not egregiously do so (Supp Fig 1), there are, for example, outliers and slight non-normality in residuals. As a result, we recommend users consider the full model output, which is provided alongside summary statistics, for their specific use-case. In future versions of quantvoe, it may be prudent to consider alternative confounder analysis strategies than a simple (mixed) linear regression, like variable selection methods (e.g. an elastic net).”

Finally, in addition to addressing the Reviewer’s specific concern above, we felt so strongly that this kind of validation was essential that we now have added a feature to the package that returns full model output for equations 10/11, allowing them to explore model summary statistics and check the fundamental assumptions of the model via plotting or other means. We have updated the README accordingly:

“Because we are using a regression analysis to look at the impact of adjusting variables, you are subject to any of the issues that could come with mixed effect (or plain linear) regression. Pay attention to the output to look for convergence warnings. Additionally, given that we provide the full model summary statistics in addition to summarized output, we recommend that the analyst also examines whether the regression is not violating any of the core assumptions of linear regression (e.g. homoscedasticity) via visualization (provided in the software). If you still have questions, feel free to contact the authors or look at the package vignette.”

And we have updated the methods in the manuscript as well:

“In addition to summarized “tidy” model statistics, we provide the full model output for Equations 10/11 as part of quantvoe, enabling users to explore model features (e.g. residuals) independently.”

1.7	There are also many different sources bias which have not been mentioned in the caveats section: quantvoe's treatment of missing variables, the effect of non-linearities, lack of account of global model fit (e.g., R2 or AIC for each vibration). The effect of non-linear terms in particular, as this will cause the number of models to increase drastically.

This is similar to a point made by Reviewer 3 point 3.2 regarding potential drawbacks of our brute force modeling strategy. We now discuss some of these points in the caveats section of the discussion. We additionally note that quantvoe returns full modeling output, so metrics like R2 can easily be extracted by the user.

We write:

“There are also potential drawbacks to our brute force modeling approach (i.e. fitting up to every possible model). For example, there may be variables not measured in a cohort that are critical for modeling, and given that random permutations of models are fit, these may be missing a certain unknown necessary adjuster. Alternatively, collider bias or reverse causality, may complicate the output of the mixed effect modeling analysis in terms of distinguishing between confounding and colliding. We also do not consider metrics such as R2 or Akaike Information Criterion (AIC) which could be used to evaluate model fit. However, full model outputs are returned as part of quantvoe, so users can analyze these metrics. Future iterations of quantvoe should automate the evaluation of models based on these and other criteria, enabling users greater clarity when interpreting our analysis.”

We additionally specifically reference non-linearities in the previous paragraph:

“Our approach, however, is not without caveats. First, our specific implementation makes use of generalized linear models, and there are many possible more complex associational methods that could potentially capture non-linearities, such as pre-transforming the variables (which itself could be thought of as yet another parameter to vary).”

1.8	Please remove 'madman' from your Github readme which is linked to from the paper as it's ableist language. Unless this has to do with the metaanalysis of gene expression datasets, in which case please clarify.

We thank the Reviewer for pointing this out and have accordingly removed this word from the README.

There are also some minor points which would aid the readability:

1.9	Please point the reader to the full definitions of the Janus effect and VoE, when they first appear. e.g., VoE is rigorously defined in lines 385 - 387, so please link to this definition.

We now provide these definitions immediately in the introduction, writing:

“Quantvoe identifies confounding in associations by a form of sensitivity analysis: modeling “Vibration of Effects,” (VoE) which is defined as how model output changes as a function of specification, specifically the distribution of the size, direction, and statistical significance of the association between two features (Fig 1B-D).[6,11–13] It is capable of identifying highly non-robust associations with “Janus Effects” (JE), where model specification yields consistently results, reporting both positive and negative output. In practice, JEs can be used to understand if an association between two variables is consistent regardless of model specification. For a full list of definitions relevant to quantvoe, please see Supp Table 1.”

We additionally provide a Supplementary Table that is a glossary of terms.

1.10	The type setting of the mathematical equations is difficult to read. Please consider using standard mathematical/statistical notation, or at least changing the font from italic to regular.

We have adjusted the fonts of all equations, as requested.

1.11	The font size of the charts is also very hard to read, please make as large as possible.

We have increased font size in all charts to ensure readability.

1.12	Make sure all acronyms are defined, e.g., TOTMETW.

We apologize for this oversight and have scoured the text, ensuring that all necessary acronyms are defined (in at least the new Supplementary Table/glossary), and those that are not necessary are removed. We specifically replaced the reference to TOTMETW in the methods.

1.13	Grammar errors: lines 55, 62.

We thank the Reviewer for catching these and have fixed them.

1.14	Inappropriate use of binary logistic regression for the vision/carrots analysis, it should be an ordinal model. No need to re-run as this was an illustrative example, but please note the reason for doing this, e.g., speed or simplicity.

We agree with the Reviewer that an ordinal model would be more appropriate. We have now updated this in the text accordingly, writing in the Methods:

“The carrot intake association was done using binary logistic regression. This is likely non-optimal and was done for the sake of simplicity, given that this was meant to illustrate just an example of VoE. Another -- more appropriate -- approach for this would be to use an ordinal model.”

**Reviewer #2:**

**[identifies himself as Florian Naudet]**

This is an important manuscript for researchers involved in the field of clinical epidemiology. This is a meta-research paper that focuses on the on of the 5 important areas of meta-research, i.e. "methods".

I support its publication, providing authors can address the following comments :

We are greatly enthused that the Reviewer finds our work important and recommends its publication. We have integrated their comments below and believe them to have greatly improved our work.

GENERAL COMMENT

2.1 I share the initial concern of the editor that the paper was not so novel. There are previous papers dealing with this concept of VoE (by the authors of this manuscript who were among the fist to identify it in epidemiology) and by other in different scientific fields (e.g. psychology etc.). However, I also appreciate that the authors are introducing a new R library and illustrate its use with some examples. I therefore consider that it has some interest/novelty. Still the decision concerning this point (novelty) can only be an editorial decision. Should the editor of PLOS Biology find it not so novel, I suggest forwarding it to another journal (e.g. PLOS MEDICINE or PLOS ONE).

We are pleased that the Reviewer sees value and novelty in our R library and the examples described in the manuscript.

To clarify the novelty of our manuscript, we now explicitly state (as Reviewer 1 also requested) how our tool operationalizes an existing framework (modeling VoE) for the first time in order to enable effective data-driven discovery in biomedical datasets.

We write in the Introduction:

“Charting a course through the process of analyzing observational data (Fig 1) is not always straightforward, however. Any decision along the analytic pipeline -- from data collection to statistical analysis -- can introduce uncertainty (e.g. false positives), obscuring underlying biology.[5–7] As a result, there is a large need for methods to aid in navigating the maelstrom of the analytic process. Here, we present a tool, “quantvoe,” for mitigating one of many potential biases: confounding via model specification. Broadly speaking, quantvoe operationalizes the identification of robust (i.e. high-confidence) vs. non-robust (i.e. low-confidence) associations, enabling greater long-term model reproducibility and interpretability. For example, if our tool had existed in the past year, we could have understood one aspect of the confusing and contradictory claims regarding COVID-19 positivity and Vitamin D levels.(Rubin 2021)”

“Quantvoe identifies confounding in associations by a form of sensitivity analysis: modeling “Vibration of Effects,” (VoE) which is defined as how model output changes as a function of specification, specifically the distribution of the size, direction, and statistical significance of the association between two features (Fig 1B-D).[6,11–13] It is capable of identifying highly non-robust associations with “Janus Effects” (JE), where model specification yields consistently results, reporting both positive and negative associations. In practice, JEs can be used to understand if an association between two variables is consistent regardless of model specification. For a full list of definitions relevant to quantvoe, please see Supp Table 1.”

And in the Discussion:

“The novelty of our tool -- quantvoe -- lies in its ability to bridge the gap between data and robust associations in observational and biomedical studies. By using quantvoe, researchers can identify which associations are robust (or not) and why this is the case prior to publishing them.”

“This software can allow researchers to automate sensitivity analyses, or Vibration of Effects, to navigate through large-scale association studies, fulfilling a tenet of biomedical data science: computing robust associations. ”

We additionally describe, briefly, how other operationalizing software has democratized existing ideas and therefore allows a field (human genetics) to be widely explored:

“Other work and software packages for modeling sensitivity under the umbrella of multiverse analysis is additionally focused less on modeling strategy and more on alternative aspects of data analysis, such as data collection.[[20]](https://paperpile.com/c/FNAJOA/uhOG4) We propose this gap is limiting the ability of researchers to navigate observational data, identifying robust associations and potential confounders. Indeed, in other disciplines (e.g. genetics) the development of analogous software (e.g. PLINK (Purcell et al. 2007)) democratized genetic epidemiology and meta-analysis (e.g. via Genome Wide Association Studies [GWASs]). Finally, there is also a need also missing from these other approaches to not just quantify VoE demonstrate how results change as a function of approach, but specifically show why model output may vary why they do: what adjusters confound associations the most and therefore should be considered in future studies.”

INTRODCUTION

2.2 I also thank the authors for providing a clear introduction to the concepts they are studying. The text is quite simple to read, there is no specific jargon and I think that it can be suitable for a large audience. For instance, the authors provide a series of examples were VoE can be at play. In their very engaging introduction, however authors refer to a paper (l.38) about "chocolate consumption and the probability of winning a Nobel Prize". Of course this paper illustrates the problems of nutritional research that can sometimes find very spurious associations. Still, I think this paper was not the best choice as it was not a very serious paper and, in my understanding, it was a paper written in a tongue in cheek way to point problems arising from ecological fallacy. I therefore suggest the use of another example.

We once again are grateful to the Reviewer for their kind words regarding our introduction, as we had worked hard to make it jargon free and interesting to individuals outside the specific field of our work. We agree, however, that upon further consideration, due to its somewhat humorous basis, the chocolate-nobel prize paper is perhaps not the best example to bring up when discussing potentially spurious correlations. As a result, we have replaced it with a more “serious” but still engaging example: red meat and heart disease risk. We now write in the introduction:

“Whether it be eyeglass usage and COVID-19 infections[1], milk and breast cancer[2], the human microbiome and any number of diseases[3], or red meat and heart disease[(Al-Shaar et al. 2020)](https://paperpile.com/c/GreDYQ/EEx8)…”

2.3 Although clear, the introduction is long and sometimes I found it a little bit repetitive and even contradictory :

. e.g. "But few of the many possible types of multiverse analysis have been fully automated and many require manual configuration." (l.67) ;

. and (l.75) "There is a need for tools to operationalize multiverse analysis through comparing model adjusting strategies."

We have now removed line 75.

. and (l.83) "However, unlike other disciplines, no standardized software package exists to systematically model VoE in massive datasets with hundreds of potential adjusting variables"

- > I therefore suggest to shorten the introduction and to clearly provide a short but exhaustive description of all tools that exist to explore VoE, by discipline and a description of the specific lack of tools in clinical epidemiology and highlight the interest of their package. I just mean that the introduction can still be a little bit simplified and more focused on the use of package among all existing initiatives in this area.

We have completely reworked the Introduction (and Discussion) in accordance with the requests of all 3 Reviewers. We have shortened the Introduction as requested. We opted to add a comparison to other tools in the Discussion in order to keep the Introduction more streamlined. While we believe a systematic review/comparison of alternative sensitivity estimation methods is outside the scope of this paper (with its novelty lying primarily in programmatically operationalizing VoE as opposed to introducing it for the first time), we hope the Reviewer will be satisfied with what we have included, which is a brief description of alternative tools and how they differ explicitly from VoE (being focused on parameter estimation instead of model comparison).

We write:

“Given these drawbacks, it is worthing noting that there are other tools, like Bayesian Modeling Averaging[47] or regularization methods (e.g. LASSO),[48] that have been used to search the space of variables measured in a dataset that predict the Y optimally; however these stand apart from our method, which automates multiverse analysis by undertaking a systematic exploration and presentation of the association and inference between an X and Y as a function of all other possible models in a dataset. Furthermore, there are other methods that could be used for sensitivity analysis. A non-exhaustive list includes VanderWeele's E-values (which measures how strong the association between potential confounding variables, the outcome, and the exposure would have to be to influence an association of interest), Monte Carlo sensitivity analysis (which attempts to correct for bias based on model parameters), or the Confidence Profile Method (which is a Bayesian approach to measure uncertainty).(VanderWeele and Ding 2017; Greenland 2005). All of these methods have their strengths and limitations(Ioannidis et al. 2019). The key difference between these tools and VoE is that VoE explicitly models the impact of specific and measured adjusting variables or model specifications on an association size instead of using outside parameters and assumptions to do so. That said, these methods could be used to augment quantvoe, or potentially they could be deployed in its stead in situations where the potential drawbacks of the brute force modeling approach (e.g., compute time) outweigh the benefits. We believe considering quantvoe alongside other tools will be crucial in the future, as its misuse or overuse would lead to an excessive on how it displays model robustness, which in turn would be its own form of bias.”

2.4 l.52 "diet" appears twice in "(e.g. age, exercise, diet, diet)".

Thank you for catching this, we have fixed this error.

RESULTS / DISCUSSION

I found this part very clear ;

2.5 I suggest that the term Janus effect (first appearance l.121 if I'm correct) can be introduced before (e.g. in the introduction) / I understand that it is detailed in the methods section but as the methods are reported at the end of the paper, it is better to introduced it before ;

In response to both this point as well as Reviewer 1’s point 1.9, we have now clearly defined Janus Effect Introduction. Please note that we additionally now provide a glossary of terms and abbreviations (Supp Table 1), also referenced in the Introduction.

2.6 In this part I would be interested to know the certainty we have a priori in the various associations explored :

. Some have an obvious causal association (i.e. anti-hypertensive agents lower blood pressure);

. Other are somewhat unlikely (e.g. Vitamin D levels affect covid, carrot etc) ;

-> I would be interested in a more systematic assessment of these priors that can help interpreting their results (authors tackle this but don't address it full l.216).

We agree with the Reviewer that further exploration of the “certainty” of various example associations is warranted to better contextualize our findings. We have updated our discussion to include these points:

“Moreover, the relationships we queried are on unequal footing in terms of causality or support in the literature, though they displayed JEs across the board. For example, the association between the anti-hypertensive Linisopril and blood pressure has a clear causal link.(Lancaster and Todd 1988) Calcium intake and bone density additionally has support in the literature, as does glucose and income (however this is not reported to be a causal relationship).(Specker 1996; Houle et al. 2016) The association between COVID and Vitamin D positivity was only reported in 2020, and is blatantly conflicting.(Rubin 2021) The components of the Framingham Risk Score, examined in Figures 3-4, each have ample support in the literature(Lloyd-Jones et al. 2004) -- though associations with them did display, in some cases, notable VoE with a range of dietary variables, indicating (as others have) that the leap between diet and coronary artery disease is not straightforward and perhaps not reliable.”

2.7 In the same spirit, I would be interested, for each association, to see the possible confounders that can be at play in the data sets that were considered (e.g. indication bias for anti-hypertensive agents).

In our discussion of potential caveats, we additionally have added in the Discussion text regarding these potential confounders for the associations queried:

“Next, given that model specification is not the only confounder that can affect results, it is of course possible that other features -- like virtues of the datasets we used in the analysis -- could be biasing our conclusions. This could be the case, for example, for the lisinopril blood pressure associations if there is indication bias. To truly explore the examples used in this manuscript, additional observational datasets, perhaps with different collection criteria, should be considered.”

2.8 As part of the question about novelty, I found it very innovative and interesting to analyse and detail the impact of each variable that could be entered in the model. I think that it point is not put forward in this paper. Still, it is very important in my opinion.

We greatly appreciate this comment and agree that the variable impact analysis extends VoE in novel directions. We have attempted to clarify this at various points in the paper:

In the Introduction:

“This analysis yields lists of candidate variables, some of which potentially could be considered as adjusters in later analyses. We specifically analyze the degree to which variables potentially impact association sizes.”

In the Discussion

“The key difference between these tools and VoE is that VoE explicitly models the impact of specific adjusters and model specifications on an association size instead of using outside parameters to do so.”

2.9 (l.209, l.210, l.211) this part of the discussion can be expanded to detail the differences between the other methods and this new one with their possible strengths and weaknesses.

In light of this comment, as well as Reviewer 1’s comments, we have extended the Discussion to include comparison to other methods and potential strengths and weaknesses.

“There are also potential drawbacks to our brute force modeling approach (i.e. fitting up to every possible model). For example, there may be variables not measured in a cohort that are critical for modeling, and given that random permutations of models are fit, these may be missing a certain unknown necessary adjuster. Alternatively, collider bias or reverse causality, may complicate the output of the mixed effect modeling analysis in terms of distinguishing between confounding and colliding. We also do not consider metrics such as R2 or Akaike Information Criterion (AIC) which could be used to evaluate model fit. However, full model outputs are returned as part of quantvoe, so users can analyze these metrics. Future iterations of quantvoe should automate the evaluation of models based on these and other criteria, enabling users greater clarity when interpreting our analysis.

Given these drawbacks, it is worthing noting that there are other tools, like Bayesian Modeling Averaging[47] or regularization methods (e.g. LASSO),[48] that have been used to search the space of variables measured in a dataset that predict the Y optimally; however these stand apart from our method, which automates multiverse analysis by undertaking a systematic exploration and presentation of the association and inference between an X and Y as a function of all other possible models in a dataset. Furthermore, there are other methods that could be used for sensitivity analysis. A non-exhaustive list includes VanderWeele's E-values (which measures how strong the association between potential confounding variables, the outcome, and the exposure would have to be to influence an association of interest), Monte Carlo sensitivity analysis (which attempts to correct for bias based on model parameters), or the Confidence Profile Method (which is a Bayesian approach to measure uncertainty).(VanderWeele and Ding 2017; Greenland 2005). All of these methods have their strengths and limitations(Ioannidis et al. 2019). The key difference between these tools and VoE is that VoE explicitly models the impact of specific and measured adjusting variables or model specifications on an association size instead of using outside parameters and assumptions to do so. That said, these methods could be used to augment quantvoe, or potentially they could be deployed in its stead in situations where the potential drawbacks of the brute force modeling approach (e.g., compute time) outweigh the benefits. We believe considering quantvoe alongside other tools will be crucial in the future, as its misuse or overuse would lead to an excessive on how it displays model robustness, which in turn would be its own form of bias.”

2.10 There is an important interest in VoE currently. This is an excellent reference and I think that the authors missed it : https://royalsocietypublishing.org/doi/pdf/10.1098/rsos.201925 / I suggest using it and discussion in depth that model specification is only one aspect of VoE ;

We thank the Reviewer for finding this reference, which we indeed did miss. We now include it in the Discussion and mention the point that VoE could potentially consider more than just model specification:

“Multiverse analyses, very broadly speaking, describe how any analytic choice (e.g. data preparation, model assumptions, model type) can change findings, and there are many different forms of them.[(Steegen et al. 2016; Orben and Przybylski 2019; Hoffmann et al. 2021)](https://paperpile.com/c/GreDYQ/Tzpg+diYx+bllu)”

2.11 I really appreciate that the authors used various examples in this paper using different databases and research question. To adopt a new tool and a new procedure, one need various examples and this is a good point here. I would however like to see more discussion about this : how many case studies may be necessary before adopting it ;

We completely agree that it is fascinating to consider how many case studies should be developed before a tool can be fully adopted.

For this manuscript we used two different datasets with large sample sizes but distinct collection methods. Within them, we aimed to demonstrate how modeling VoE can be useful for a number of different applications across observational data science, from dietary epidemiology to COVID associations to drug efficacy to the impact of socioeconomic status on health.

However, the Reviewer raises an excellent point -- when do we “know” that the software is ready to be fully adopted as a function of case studies? Are case studies even the right metric? We believe it is impossible to say when this inflection point happens, and further we claim that it is often field-specific. For example, we write in the manuscript that all the associations should likely be tested in additional datasets to confirm the presence of VoE:

“To truly explore the examples used in this manuscript, additional observational datasets, perhaps with different collection criteria, should be considered.”

However, instead of relying on the number of case studies to demonstrate a software’s readiness for deployment, we instead in this paper claim we have shown through unit testing and examples that quantvoe generates reliable and interpretable results.

Further, our approach to software development enables rapid modification if needed in the future. To complete the revisions for this manuscript, we had to update our package in a number of small ways (e.g. reporting full model output from the confounder analysis). This was easy, because we use the Agile framework for rapid design, development, and testing.

As another example, we additionally have a separate manuscript in review at PLOS Biology using quantvoe to explore the robustness of nearly 600 associations in the microbiome space. This is a completely different field, however we believe our work shows quantvoe’s specific relevance to the microbiome despite being only one “case study”. That said, if Reviewers ask us to extend our package for our microbiome paper, we will do so with ease due to our Agile software development framework.

Therefore, given our software development paradigm as well as the diversity of examples used in this paper we claim our tool is ready to be adopted now. However, we are perfectly aware that it may need to be further tailored for future applications, and its use in a completely new field should be done with the utmost rigor (as one would with any tool).

2.12 And importantly I would expect more discussion on "How can one use this framework in a practice ?" If it was badly used, this extreme form of sensitivity analysis could be a massive destruction weapon for the field of epidemiology. If correctly and smartly used, I'm sure that it can increase value of the field. Can authors elaborate a little bit on this point ?

We agree this is a point well worth making to avoid careless and detrimental deployment of VoE and that it should be used alongside other tools down the line. We now discuss this in the Discussion, writing:

“That said, these methods could be used to augment quantvoe to better inform its use, or potentially they could be deployed in its stead in situations where the potential drawbacks of the brute force modeling approach outweigh the benefits. We believe considering quantvoe alongside other tools will be crucial in the future, as its misuse or overuse would lead to an excessive on how it displays model robustness, which in turn would be its own form of bias.”

METHODS

This is very clear. Only two points :

2.13 Please specify if this study was registered or not (sorry if I missed it). I always think that pre registration is a good thing, even for this kind of methodological research. If it was not pre-registered, I would like to see this explicitly acknowledged and to read the reasons for this.

We agree in retrospect that we should have pre-registered this study. However, we did not at its conception, mostly due to the fact that we did not classify it as a “hypothesis generating” or “hypothesis testing” study outright, considering it more methodological development, as the Reviewer points out. Upon further investigation, though, we do agree with the Reviewer that pre-registration may have been appropriate. We apologize for this oversight.

2.14 Last, I think that some points can be moved in the web appendix (e.g. instructions to download the package) but this is rather a suggestion.

We thank the Reviewer for this comment, as it forced us to look hard at our methods. We appreciate that they are long, and in the process of responding to reviews we did search for places to streamline. However, we hesitate to move too much out, as we would rather arm readers with as much information as possible in order to best use quantvoe.

**Reviewer #3:**

Charting a course for robust discovery in biomedical data science

This paper restate and reinforce the communication of the so called "vibration effect", "multiverse", "Analytical flexibility", etc in a general context.

3.1 The term vibration of effect should be clearly positioned compared to previous references.

We now clearly position this term in the Introduction, alongside previous references. We additionally include a description of all relevant terms (such as vibration of effects) in a glossary, now listed in a Supplemental Table (1). We write, in the introduction:

“Quantvoe identifies confounding in associations by a form of sensitivity analysis: modeling “Vibration of Effects,” (VoE) which is defined as how model output changes as a function of specification, specifically the distribution of the size, direction, and statistical significance of the association between two features (Fig 1B-D).[6,11–13] It is capable of identifying highly non-robust associations with “Janus Effects” (JE), where model specification yields consistently results, reporting both positive and negative output. In practice, JEs can be used to understand if an association between two variables is consistent regardless of model specification. For a full list of definitions relevant to quantvoe, please see Supp Table 1.”

3.2 The brute force approach described should be clearly related to variable selection, variable importance, and multiple comparison statistical techniques in the introduction. If scientists are to use the package to decide on the presence of an association, the reporting should consider the factors influencing the set of results. In particular, improvement or loss of measured association should be predictable through estimation of the linear dependency of confounders with the dependent variable.

As noted by the authors, the number of dependent variables can be high and strategies to account for these cases should be better described.

We now clarify in the Discussion and Methods the potential drawbacks to (and their potential solutions) to this brute force approach. We emphasize that while the current software can randomly sample variables to include, this may not be optimal in the long term and that future effort (we claim outside the scope of this current work) should be exerted to determine a mathematically justifiable minimum set of equations/variables to test given a dataset. In this vein, we write in the Discussion:

“There are also potential drawbacks to our brute force modeling approach (i.e. fitting up to every possible model). For example, there may be variables not measured in a cohort that are critical for modeling, and given that random permutations of models are fit, these may be missing a certain unknown necessary adjuster. Alternatively, collider bias or reverse causality, may complicate the output of the mixed effect modeling analysis in terms of distinguishing between confounding and colliding. We also do not consider metrics such as R2 or Akaike Information Criterion (AIC) which could be used to evaluate model fit. However, full model outputs are returned as part of quantvoe, so users can analyze these metrics. Future iterations of quantvoe should automate the evaluation of models based on these and other criteria, enabling users greater clarity when interpreting our analysis.”

3.3 It seems to this reviewer that the key aspect is wether reporting the number of positive association compared to negative association is reasonable in general (Janus effect). Consider the case of one confounder is highly correlated with the independent variable, this would lead to 2^(p-1) models with very poor association (p is the number of dependent variables), this number would rapidely increase with the number of covariate related to the independent variable. However, this would assume that it makes sense to include these covariates, which is a strong assumption of the proposed framework. The context in which this type of brute force analysis make sense should be therefore be emphasized in the introduction as well as in the discussion, since linear models are generally interpreted as causal models.

We agree this is a potential drawback of our analysis (and indeed all existing VoE analysis). We now describe this clearly in the text. We further integrate this point into comments by Reviewer 2 and Reviewer 1 regarding comparison to other techniques, using the logic described by Reviewer 3 above as a potential example of when comparison to another sensitivity analysis method may be warranted.

We write:

“Our approach, however, is not without caveats. First, our specific implementation makes use of generalized linear models, and there are many possible more complex associational methods that could potentially capture non-linearities, such as pre-transforming the variables (which itself could be thought of as yet another parameter to vary). Second, our definition of Janus Effect is only contingent on association size, and not statistical significance. An alternative approach would be to measure Janus Effects only for estimates that passed a certain significance cutoff. We deliberately chose to avoid this as 1) p-values vary highly depending on small changes made by the researcher and significance thresholds are, at best, somewhat subjective and 2) while approaches exist for inference after feature selection in machine learning (e.g., “post-selection inference”[50]), it is still not straightforward to estimate inferential statistics on many popular modeling approaches like random forests or similarly complex machine learning methods.

There are also potential drawbacks to our brute force modeling approach (i.e. fitting up to every possible model). For example, there may be variables not measured in a cohort that are critical for modeling, and given that random permutations of models are fit, these may be missing a certain unknown necessary adjuster. Alternatively, collider bias or reverse causality, may complicate the output of the mixed effect modeling analysis in terms of distinguishing between confounding and colliding. We also do not consider metrics such as R2 or Akaike Information Criterion (AIC) which could be used to evaluate model fit. However, full model outputs are returned as part of quantvoe, so users can analyze these metrics. Future iterations of quantvoe should automate the evaluation of models based on these and other criteria, enabling users greater clarity when interpreting our analysis.

Given these drawbacks, it is worthing noting that there are other tools, like Bayesian Modeling Averaging[47] or regularization methods (e.g. LASSO),[48] that have been used to search the space of variables measured in a dataset that predict the Y optimally; however these stand apart from our method, which automates multiverse analysis by undertaking a systematic exploration and presentation of the association and inference between an X and Y as a function of all other possible models in a dataset. Furthermore, there are other methods that could be used for sensitivity analysis. A non-exhaustive list includes VanderWeele's E-values (which measures how strong the association between potential confounding variables, the outcome, and the exposure would have to be to influence an association of interest), Monte Carlo sensitivity analysis (which attempts to correct for bias based on model parameters), or the Confidence Profile Method (which is a Bayesian approach to measure uncertainty).(VanderWeele and Ding 2017; Greenland 2005). All of these methods have their strengths and limitations(Ioannidis et al. 2019). The key difference between these tools and VoE is that VoE explicitly models the impact of specific and measured adjusting variables or model specifications on an association size instead of using outside parameters and assumptions to do so. That said, these methods could be used to augment quantvoe, or potentially they could be deployed in its stead in situations where the potential drawbacks of the brute force modeling approach (e.g., compute time) outweigh the benefits. We believe considering quantvoe alongside other tools will be crucial in the future, as its misuse or overuse would lead to an excessive on how it displays model robustness, which in turn would be its own form of bias.”

3.4 Implementation: the package seems to be lacking a unit testing suite.

We have now made more accessible our testing suite and added to its documentation in the GitHub repository. We additionally have further highlighted the package vignette. For a greater description of changes in additions, please see the README, Reviewer 1’s point 1.3 as well as the section immediately below this one.

**Details on unit testing suite regarding Reviewer 1 point 1.3 and Reviewer 3 point 3.4.**

In addition to the details we have provided in the point by point response above regarding the testing suite, example code, and vignette, we will demonstrate here, including screenshots for reference, deploying our testing on a fresh install of the package. This is to indicate where the specific datasets are and that the testing suite is present and functional.

Please note that we ran this process on 3 different compute environments (i.e. 2 different local machines as well as a remote cluster). We only display one here for reference.

We additionally include these screenshots in the testthat folder in the GitHub for users who want to visually see examples of running testing. We have additionally mentioned this folder in the README.

As seen above, all 9 tests (the output and structure of each are described in the point by point responses) complete successfully. This was the case on all machines tested.

For the added benefit of the Reviewers, we additionally are including in this document what the example code looks like when run locally (in the beginner, intermediate, and advanced examples).

EXAMPLE 1

EXAMPLE 2

EXAMPLE 3

We hope that these details have clarified the existence, presence, and running strategy for our testing suite, and we thank the Reviewers for motivating us to make it clearer.
